# Significant Change Spotting for Periodic Human Motion Segmentation of Cleaning Tasks Using Wearable Sensors

**DOI:** 10.3390/s17010187

**Published:** 2017-01-19

**Authors:** Kai-Chun Liu, Chia-Tai Chan

**Affiliations:** Department of Biomedical Engineering, National Yang-Ming University, 155, Li-Nong Street, Section 2, Peitou, Taipei 11221, Taiwan; g30104026@ym.edu.tw

**Keywords:** human motion segmentation, wearable sensors, activities of daily living, automatic activity monitoring

## Abstract

The proportion of the aging population is rapidly increasing around the world, which will cause stress on society and healthcare systems. In recent years, advances in technology have created new opportunities for automatic activities of daily living (ADL) monitoring to improve the quality of life and provide adequate medical service for the elderly. Such automatic ADL monitoring requires reliable ADL information on a fine-grained level, especially for the status of interaction between body gestures and the environment in the real-world. In this work, we propose a significant change spotting mechanism for periodic human motion segmentation during cleaning task performance. A novel approach is proposed based on the search for a significant change of gestures, which can manage critical technical issues in activity recognition, such as continuous data segmentation, individual variance, and category ambiguity. Three typical machine learning classification algorithms are utilized for the identification of the significant change candidate, including a Support Vector Machine (SVM), k-Nearest Neighbors (kNN), and Naive Bayesian (NB) algorithm. Overall, the proposed approach achieves 96.41% in the F1-score by using the SVM classifier. The results show that the proposed approach can fulfill the requirement of fine-grained human motion segmentation for automatic ADL monitoring.

## 1. Introduction

During the past decades, there has been extraordinary improvements in public health and medicine, allowing people to live longer and healthier. The proportion of the aging population is rapidly increasing around the world [1,2]. Aging brings many challenges to the elderly due to their cognitive decline, chronic age-related diseases, as well as limitations in physical activity, vision, and hearing. Moreover, multiple chronic diseases can limit daily functioning and hinder independent daily living [3]. Therefore, the early detection of meaningful change in aging is fundamentally important for timely prevention and treatment. The measurement of daily functionality through the activities of daily living (ADL) monitoring has become very important work in the assessment of functional status of the elderly. Traditionally, approaches for measuring ADL performance and assessment of clinically meaningful change are based on questionnaires, interviews, and in-person visits. However, it often has poor demarcation as to onset and transition to new states. The decline of ADL performance progress is slow over time, making the changeover to a new state difficult to recognize. These approaches are also hard to continuously identify the full range of potential events or trends of interest.

In recent years, advances in microelectromechanical systems (MEMS) and information and communications technology (ICT) have created new opportunities for automatic ADL monitoring to improve the quality of life and provide adequate medical service for the elderly. Several well-known approaches have been applied to the development of automatic ADL monitoring systems, such as ambient-based and wearable-based approaches. Ambient-based ADL monitoring systems refer to the practice that diversity of multimodal sensors, actuators, and devices are installed in the environment, which can recognize and inference individual’s ADLs by the detection of interactions between the individual and the environment. Most of the ambient-based approaches detect the environmental change based on sensor events. For example, using infrared sensors placed on doors to detect room transitions [4], or using RFID reader sensors to trace object usage [5]. Some works install large sensors in the home environment to monitor ADL performance [6]. However, some applications include, e.g., the monitoring of specific motion in fluid intake [7], or the classification of dietary intake gestures [8,9] for an automated ADL monitoring system, where reliable human motion on a more fine-grained level is needed, especially for hand gesture spotting. Wearable-based ADL monitoring systems are considered one of the most prevalent approaches to capture and track human motion during ADL tasks. The attributes of wearable sensors, such as high computational power, small size, and low cost, allow people to wear them over extended periods of time. Wearable sensors can be embedded into clothes, eyeglasses, belts, shoes, watches, and mobile devices to collect various information about ADLs, such as human motion, vital signs, and context information. However, many technical challenges arise from the fact that there is a considerable amount of variability inherent in individual ADL patterns if there is no manual intervention or prior knowledge.

The continuous monitoring of particular human motion during daily living can provide a measurement of daily functionality for remote health monitoring applications, especially in early detection of a change in aging. Analysis of the specified gestures or motions can provide the clinical professional with specific ADL performance metrics, such as the number of repetitions performed and the duration of each repetition, many of which are hard to monitor by current assessment strategies. Automatic ADL monitoring requires reliable ADL information on a fine-grained level, especially for the assessment of daily functionality in remote health monitoring applications, especially in early detection of a change in aging. To enable continuous monitoring and measurement, the ADL monitoring system has to segment and classify specific gestures and motions from continuous data. The accuracy of the segmentation approach affects the ability and granularity of the monitoring system. Previous work also shows that the performance of the best classifier sharply decreases when the segmented data are non-differentiable [10]. Therefore, some critical issues in data segmentation techniques need to be dealt with. Two common technical challenges are identified as follows:

(1) Since the data stream obtained from the sensor is continuous and unsegmented, there is no idea about the numbers of human motions have actually been performed, while ADLs last for a period of time;
(2) Human motions can be performed slowly or quickly, which causes all motions during ADL performance to be full of variance even for the individual.

In this work, we propose a significant change spotting mechanism for periodic human motion segmentation during cleaning task performance. A novel approach is proposed based on the search for significant change of human motion, which can manage critical technical issues in segmentation techniques, such as continuous data segmentation, individual variance, and category ambiguity. Three different classification algorithms are utilized for the identification of the significant change candidate, including a Support Vector Machine (SVM), k-Nearest Neighbors (kNN), and Naïve Bayesian (NB) algorithm. The proposed approach for cleaning task monitoring can provide an example of the feasibility in daily living based on a recording from a healthy adult. Additionally, the proposed approach is suitable for dealing with the high dimensionality signals of hand motion, and explicitly models both spatial and temporal variability to provide accurate segmentation results. The rest of the paper is organized as follows: we briefly introduce related work in Section 2; in Section 3, the novel significant change spotting approach is proposed to provide robust and adaptive human motion segmentation for cleaning task monitoring, which is based on search and spotting of significant changes during cleaning task performance; the experimental results validate the capabilities of our proposed mechanism are addressed in Section 4; in Section 5, we discuss the limitations of the current work and potentiality of future work; and, finally, the conclusion is presented in Section 6.

## 2. Related Work

ADL recognition using wearable sensors is still an open research area that involves a sequence of signal processing, pattern recognition, and machine leaning techniques. Generally, the framework of deciphering ADLs from the sensing data contains data acquisition, signal preprocessing, data segmentation, feature extraction, and classification processes. For an activity recognition system, data acquisition is the input that collects a sequence of data using various types of sensors, such as inertial sensors [7,8,11], microphones [12], and temperature and altimeter sensors [13]. Various filters utilized in signal preprocessing, including high-pass [14,15], low-pass [14,16,17], and common median [17,18], are deployed in the removal of high-frequency noise or low-frequency artifacts. The preprocessed data are split into a number of chunks in data segmentation. Later, feature extraction derives salient and distinguishable activity characteristics from the segmented data. Common feature extraction involves analyzing heuristic, time-domain, frequency-domain, and time-frequency properties of the segmented data. The explanation of filters for wearable-based human motion recognition has been explored in [13,18,19]. Finally, machine learning or pattern recognition approaches are deployed for activity classification.

There have been a number of machine learning techniques used in activity recognition, such as Support Vector Machine [13], k-nearest neighbors [20], Naive Bayesian [21], and Decision Trees [22]. Since the information of hand movements has a high temporal content, time sequence analysis methods are widely used for hand movement recognition. The most commonly used technique are Hidden Markov Models (HMM) [23], which are an underlying model, and a stochastic Markovian process that is not directly observable. The hidden nodes represent activities and the observable nodes represent combinations of the selected features. The probabilistic relationships between hidden nodes and observable nodes and the probabilistic transition among the hidden nodes are estimated through the relative frequency with which these relationships occur in the observed data. Given input series data, the HMM finds the most likely sequence of hidden states, or activities, which could have generated the observed activity. Alternatively, template matching is a well-known approach for the comparison of time-series data, which is widely utilized in pattern recognition. Stiefmeier et al. [24] proposed an approach based on trajectory calculation and aggregation to transmit the continuous sensor data into motion strings, then using a Dynamic Time Warping (DTW) algorithm to match and classify the observed patterns. The DTW algorithm uses a time-series similarity measure for sequential pattern matching. DTW-based classification minimizes the effects of shifting and distortion by allowing elastic transformation of time series, which is a benefit to recognizing hand movements with different durations in the real world. However, some technical challenges should be tackled, such as the template selection, threshold configuration, and observed segment extraction while spotting gestures in an unconstrained daily living scenario. Since in this work we aim to deal with the issues in human motion segmentation, the majority of related works are introduced below.

Traditionally, the concept of representing the raw data in segmented forms is to provide a concise display and clear insight into their basic characteristics through the appropriate approximation forms, with minimal loss of relevant information. For example, Keogh et al. [25] proposed Sliding Windows, Top-Down, Bottom-Up, and Sliding Windows and Bottom-Up (SWAB) segmentation techniques using Piecewise Linear Representation (PLR) to approximate data and find segmentation points. Most of these works are aimed at minimizing fit error and optimizing the user-specified threshold. However, the data segmentation process has been performed in various ways according to the requirements of the applications and the sensor types.

There are three common categories of segmentation approaches for processing of sensor data, illustrated as follows. The first approach is to divide a sensor data sequence into a fixed-size interval using a sliding window algorithm, for example, Banos et al. [26] explored the different window size (from 1 to 7 s) impact on physical activity recognition using a body-worn sensor. Each of the resulting partitions shares the same time interval, known as the sliding window technique. The technique has the advantage of reducing computation complexity for an activity recognition system. However, only using a fixed-size window during activity recognition might cause the activity recognition system to be incapable of dealing with diversified ADLs. Another approach is to rely on known activity patterns to segment sensor data explicitly. For example, Amft et al. [27] adopted a template-based approach that utilized multiple detectors to search for a potential activity event in a determined boundary, which are manually annotated events and segmentation points. However, if the search boundary is too wide, that adds to the computational complexity.

To improve on the drawbacks of the aforementioned approaches, the activity-defined and event-defined segmentation approaches are utilized to segment successive data. The activity-defined segmentation approach divides the sensor data into a number of chunks based on the detection of activity changes. For example, Sekine et al. [28] proposed a model based on wavelet decomposition to detect frequency changes for three walking activities (level walking, walking upstairs, and walking downstairs) from a continuous record. Various works formulate changes in signal variance as a cost function for determining segment points using a neural network [29], probabilistic approach [30,31,32,33,34,35], and rule-based approach [36]. 

An alternative segmentation approach for activity recognition is the event-defined segmentation approach, which consists of locating specific events and defining successive data partitioning. Gait analysis has principally benefited from this type of segmentation approach. Some specific events are utilized to find the gait cycle; for example, the detection of the initial and end contact of the foot with the ground are recognized through analyzing the foot’s linear acceleration [37]. They have proved such segmentation approaches in many specific applications can achieve good performance of segmentation and recognition. Since ADLs are more complicated than some gestures or activities in specific applications, such as gait cycle analysis and rehabilitation exercise, the existing approaches are limited to some technical issues while the target activities are performed in daily living. The first is that the determination of specific events for motion segmentation and spotting is a difficult issue as the sensing signal collected from human body shows high dimensionality. Secondly, ambiguity is the common issue for most of the existing event-defined segmentation approaches, which focus on the detection of a trigger point with semantic meaning. Detection of peaks [38] and zero-velocity crossing [39] are the efficient segmentation approaches to characterize significant events, while these segmentation approaches only work with certain types, but not all, motions during ADLs. Several factors, including individual habit, the variability of movement velocity, high dimensionality of the sensing signal, and the abundance of activity types lead the specific event to having different characteristics. Finally, event-defined segmentation approaches based on the trigger point suffer from the situation of under-segmentation or over-segmentation, and tend to generate large numbers of false positives.

While most of the previous works focus on the identification of the “point” to segment activity, the proposed segmentation approach tackles these weaknesses and provides a solution that is adaptive and robust against temporal and spatial variations, with the capability of dealing with the situation of under-segmentation or over-segmentation.

## 3. Proposed Segmentation Approach

In the performance of daily cleaning tasks, an activity scenario is an event or series of actions and gestures, including spontaneous and periodic human actions. The periodic human actions in this work are composed of motions, but with different directions, forces, and time intervals. For example, the participant performed, 10 times, cleaning motions during a window cleaning task, where each cleaning activity can be performed in up, down, forward, and backward directions according to the cleaning tasks. Obviously, the change of direction can be a significant event to gather the human motion segment from continuous sensing data. In order to detect the significant event for accurate segmentation from continuous cleaning data, there are two main considerations of the proposed approach for human motion segmentation. Firstly, rather than segmenting cleaning motion based on detection of a trigger point, the proposed significant change spotting is to observe a significant series from continuous cleaning motion. A significant series can be defined as an interval of series sensing data that indicates the human applied force to change the direction. Secondly, to reduce computation complexity in the presence of high dimensionality data, the segmentation and spotting mechanism should be as computationally efficient as possible. A series of processes for significant change spotting has been employed, such as dominant axis identification, event detection, and feature analysis.

**Figure 1:**
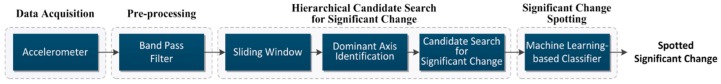
The architecture of the proposed approach for the segmentation mechanism, including data acquisition, pre-processing, hierarchical candidate search for significant change, and significant change spotting.

**Figure 2:**
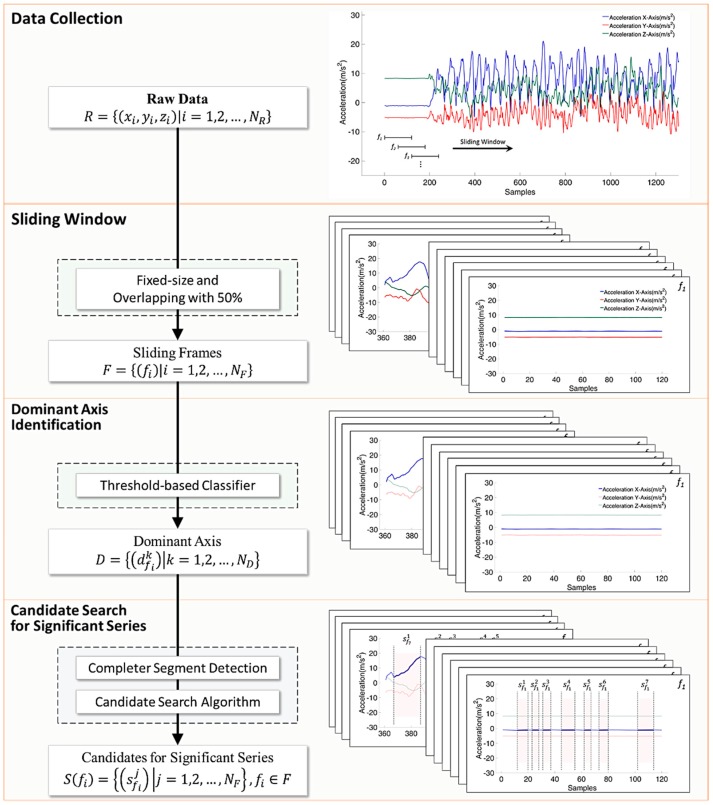
Framework of the hierarchical candidate search for significant change from raw data.

Figure 1 shows the architecture of the proposed system. It consists of four processes: data acquisition, pre-processing, a hierarchical candidate search for significant change, and significant change spotting. In the data acquisition process, the data collects sensing data from acceleration, and the band pass filters are utilized for all sensing data. The choice of activity of daily living, experimental setup, and pre-processing are described in respective subsections. As shown in Figure 2, a hierarchical candidate search for significant change is the two-stage process that searches all possible candidates for significant change spotting based on the observation of a significant series during cleaning motion performance. Finally, feature analysis and machine learning-based spotting are executed for the process of significant change spotting.

### 3.1. Data Acquisition and Pre-Processing

#### 3.1.1. Choice of Activity of Daily Living

There are two main types of ADLs, namely basic ADLs (BADLs) and instrumental ADLs (IADLs). The BADLs are activities necessary for self-care, while IADLs are not. Examples of BADLs are feeding, bathing, dressing, grooming, etc. IADLs, such as using telephone, housework, doing laundry, etc. In previous works, ADL monitoring systems have been developed for five common ADLs from the Barthel Index, including feeding, grooming, dressing, mobility, and stair-climbing [40]. Some works focus on the specific measure of a particular interest in mobility performance [41]. However, IADL assessments are more suitable than BADL assessments for the early detection of daily functional decline [42]. This work aims to segment cleaning motions (e.g., wiping, sweeping, and mopping) during cleaning tasks, which is one of the general IADL categories to assess the self-care and independent living capability [43].

#### 3.1.2. Experiments Setup and Pre-Processing

**Figure 3:**
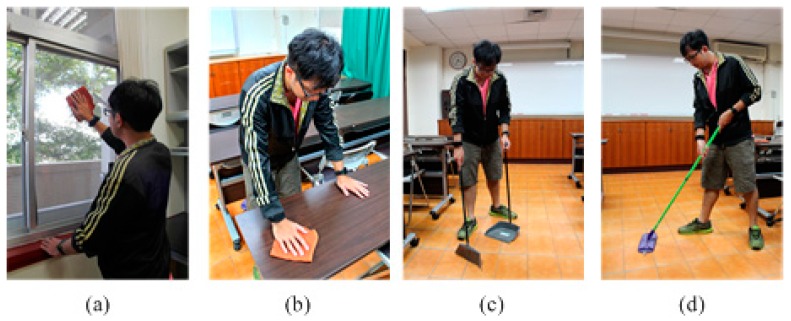
(**a**) Clean Window; (**b**) Clean Table; (**c**) Sweep Floor; (**d**) Mop Floor.

We conducted an experiment on six subjects involving four males and two females, aged from 23 to 29 years, who performed four cleaning tasks, included cleaning a table, cleaning a window, sweeping a floor, and mopping a floor, the details of which are described in Table 1 and Figure 3. All cleaning tasks are constrained to an area of 5.5 m × 6.2 m for the purposes of capturing video of the session. The targets of cleaning tasks, such as the window and table are 1.5 m × 1 m and 1.2 m × 0.7 m. Each subject is asked to perform a series of cleaning tasks for five days over two weeks. The cleaning tasks was repeated or sustained for a period and no time constraints, but marked with the start and stop times, and labeled with the name of each task. The overall dataset covers 112.4 min.

**Figure 4:**
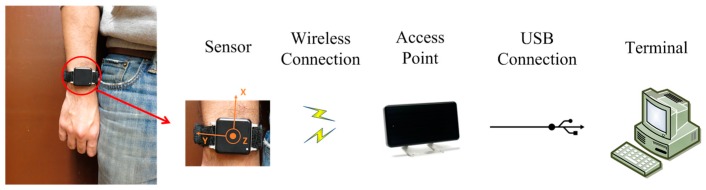
Illustration of sensor placement and the hardware platform.

The wearable sensor named Opal (APDM, Inc., Portland, OR, USA) is utilized to record sensing data of hand movements in this work. The dimension and weight of the sensor node are 48.5 × 36.5 × 13.5 mm and weigh 22 g. The wearable sensor includes a triaxial accelerometer and transmits sensing data wirelessly through an RF module. The sampling rate is 40 Hz, the range is ±6 g, and battery life of the sensor allows eight hours of continuous data-logging. The sensor placement usually depends on the target application and motions as different sensors provide different information which may reflect more important information in specific applications than others. The sensor of this work is attached to the right wrist since the execution of cleaning tasks has a close relationship to the performance of the upper limb. Furthermore, wrist-worn sensors have the attribute of low-intrusiveness, making them suitable for continuous collection of motion data in daily living. Then, an access point receives the acceleration wirelessly from the sensor nodes and transmits the sensing data to the terminal through a USB connection, as shown in the Figure 4. A third-order Butterworth filter, having cut-off frequencies of 0.1 Hz and 12 Hz is applied to the raw data in the process of pre-processing, in order to remove the artefacts and noise [18,44,45].

### 3.2. Hierarchical Candidate Search for Significant Change

As shown in Figure 2, firstly, the sliding window approach with 50% overlapping is applied to divide the sensor data into chunks, where the window size is four seconds. The setting provided a sufficient buffer to cover at least one complete human motion as the longest duration of complete human motion does not exceed two seconds in this work. Each chunk is a sliding buffer and possibly contains several gestures, which provides a global view of the continuous sensor data. Then dominant axis identification is executed for each of the sliding frames. Finally, a candidate search algorithm for a significant series is proposed to detect the events indicating direction changes of gestures. The detail of the dominant axis selection and candidate search algorithm for a significant series are described in the following subsections.

Formally, let a sequence of raw sensing data $R=\left\{\left(x_{i},y_{i},z_{i}\right)|i=1,2,\ldots ,N_{R}\right\}$ be divided into sliding frames $F=\left\{\left(f_{i}\right)|i=1,2,\ldots ,N_{F}\right\}$ with a defined window size. Secondly, dominant axis identification is applied to each sliding frame and obtains a vector of the dominant axis for a significant series search $D=\left\{\left(d_{f_{i}}^{k}\right)|k=1,2,\ldots ,N_{D}\right\}$. Finally, the process of the candidate search for significant change is applied to any sliding frame and gathers a vector of candidates for significant change spotting $S\left(f_{i}\right)=\left\{\left(S_{f_{i}}^{j}\right)|j=1,2,\ldots ,N_{F}\right\},f_{i}\in F$.

#### 3.2.1. Dominant Axis Classifier

In order to improve the proposed algorithm’s robustness and computational complexity in the presence of high-dimensionality data, a dominant selection is designed for each sliding window. The force of motion tends to focus on a particular direction during cleaning task performance, thus, it can be assumed that the cleaning motion undergoing the significant change of axis is the dominant one. The axis with the highest difference among all axes is assumed to be dominant in the sliding frame. The dominant axis can be identified by a threshold-based identifier. Given that an existing sliding frame $f_{\alpha }$ belonging to F and with a total of three axes from triaxial acceleration x, y,z, the dominant axis can be identified by Equation (1):

(1) $$\underset{x,\;y,\;z}{\mathrm{argmax}}\;DAI\left(x,\;y,z\right)=\left\{d_{i}=Avg_{max}^{i}-Avg_{min}^{i}|i=x,y,z\right\}$$

where $Avg_{min}^{i}$ and ($Avg_{max}^{i}$) are the average minimum (maximum) value, and defined as the average of the first smallest (largest) ten percent of values in the axis i of the sliding frame. The value of the axis in the sliding window with the maximum is identified to be dominant for the current sliding frame.

#### 3.2.2. Hierarchical Candidate Search for Significant Series

**Figure 5:**
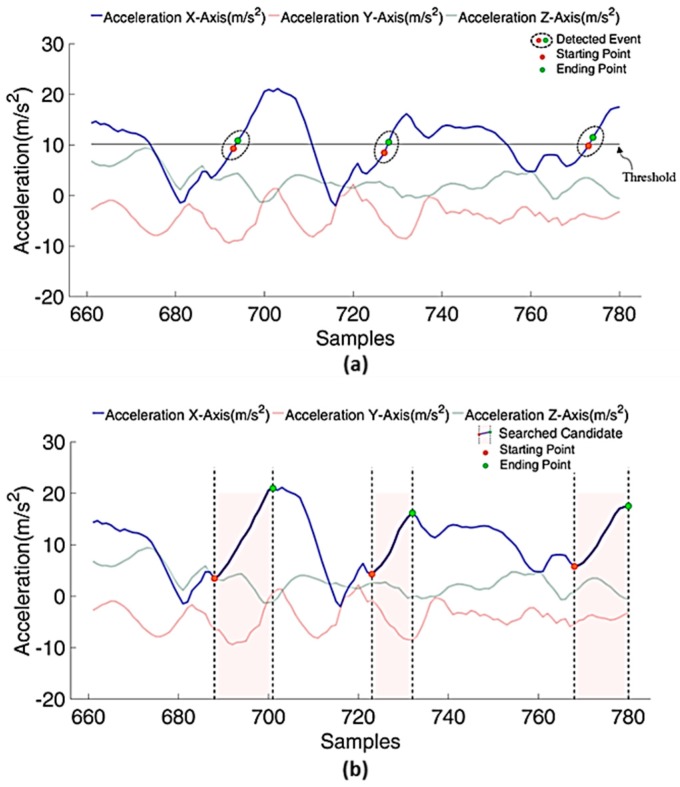
Tri-axial acceleration of a subject performing floor mopping. (**a**) The threshold is determined by the dominant axis (acceleration of the X-axis). Each circle denotes the detected event, which contains starting and ending points, and crossing the threshold in ascending order. (**b**) The covered region by the starting and end points denote the searched candidate for significant change spotting.

**Figure 6:**
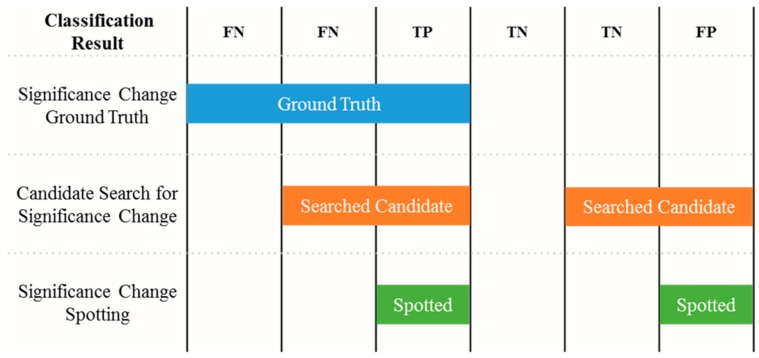
From bottom to top, the spotting process includes a candidate search for significant change and significant change spotting. Finally, the spotted candidates are compared against to the ground truth in terms of the classification result. If there are no searched candidates, the output of cleaning gesture must be negative.

Since hand motion in cleaning tasks is more complicated than ambulatory movements, the improved search algorithm for a significant series is proposed in this work. The pseudocode of the search algorithm is described in Algorithm 1, and the illustration of the proposed candidate search for a significant series is shown in Figure 5. Traditionally, the exemplar motions are scanned firstly for key features or events, such as peaks, zero crossing points (ZCPs), or mean crossing points (MCPs) in velocity or acceleration as a way to approximate a potential segmentation region in the sliding frame. Previous works also show that using velocity features allows the segmentation algorithm to spot the general shape of the interested motion more robustly than approaches that rely on distance measures [22]. As shown in Figure 6a, the detection of MCPs is adopted in the proposed segmentation approach. Firstly, the threshold is defined as the average of sensing data in the dominant axis (for Line 1). Then detection of the MCP during human motion is determined by events composed of two successive data, which cross the threshold in ascending order (for Lines 2–7). These detected events are assumed as potentially part of the significant series. Instead of using a trigger point for the segmentation, this work focuses on spotting a significant series as the transition region for the human motion segmentation. However, the significant series are not always performed as the monotonic series since motions can be performed in various ways. Much of the noise, such as local peaks and noise spikes, that occurs in the signal might be caused by the vibration of muscle, individual habit, and the variability of movement velocity. In order to tackle the issues to accurately locate the starting and ending points of a complete candidate, the extension function is applied to all events (for Lines 8 and 13).

As shown in Figure 5a, for any detected event, one of the data points with the smaller value is considered as the initial starting point of candidate, and the other one with the larger value is considered as the initial ending point of candidate. In the following, the searching end point for the candidate is introduce firstly. If the significant point with the maximum value in the current extension is equal to the initial ending point or searched significant point in the previous extension, the initial ending point or searched significant point is identified as the ending point of a complete candidate, and the algorithm stops searching for the ending point of candidate. Suppose $d_{initial\_end}$ is the initial ending point of detected event before extension, l is the number of times of extension during the ending point search with the initial value equal to 1, $\alpha_{upper}$ is the upper boundary of each time extension, and the ending point $d_{end}$ of the candidate can be found in $N_{total\_end}$ times of extension. The extension function $E_{end}$ is applied to search for the ending point of the candidate, which can be defined as followed:

(2) $$d_{end}=E_{end}\left(d_{initail\_end},\alpha_{upper},\;l\right)\;=\{\begin{array}{cc} d_{initial_{end}}, & x_{l}=d_{initial_{end}}\\ E_{end}\left(x_{l},\alpha_{upper},\;l+1\right), & x_{l}\neq d_{initial\_end} \end{array},$$

(3) $$x_{l}=argmax\left(d_{initail\_end},d_{initail\_end+1},\ldots ,d_{initail\_end+\alpha_{upper}}\right),$$

where $1\leq l\leq N_{total\_end}$, $d_{end}$ is the ending point of the candidate, and $x_{l}$ is the significant point with the local maximum in the $l_{th}$ extension, which can be defined in Equation (3).

In the following, the searching starting point for a candidate is introduced. If the significant point with the minimum value in current extension is equal to the initial starting point or searched significant point in the previous extension, the initial ending point or searched significant point is identified as the starting point of the complete candidate, and the algorithm stops searching for the starting point of the candidate. Suppose $d_{initial\_start}$ is the initial starting point of a detected event before extension, p is the number of times of extension during the starting point search with the initial value equal to 1, $\alpha_{lower}$ is the lower boundary of each time extension, and the starting point $d_{start}$ of the candidate can be found in $N_{total\_start}$ times of extension. The extension function $E_{start}$ is applied to search starting point of the candidate, which can be defined as followed:

(4) $$d_{start}=E_{strat}\left(d_{initail\_start},\alpha_{lower},\;p\right)=\{\begin{array}{cc} d_{initail\_start}, & x_{p}=d_{initial\_start}\\ E_{strat}\left(x_{p},\alpha_{lower},p+1\right), & x_{p}\neq d_{initial\_start} \end{array},$$

(5) $$x_{p}=argmin\left(d_{initail\_start-\alpha_{lower}},d_{initail\_start+1},\ldots ,d_{initail\_start-1},d_{initail\_start}\right)$$

where $1\leq l\leq N_{total\_start}$, $d_{start}$ is the starting point of the candidate, and $x_{p}$ is the significant point with the local minimum in the $p_{th}$ extension, which can be defined in Equation (5).

### 3.3. Significant Change Spotting

In this subsection, the applied features extraction and selected machine learning-based classifier are introduced. A machine learning-based classifier is used to identify whether the candidate is really a significant or non-significant series. Three typical machine learning algorithms are selected as the core technique of a machine learning-based classifier for a performance comparison of the proposed segmentation mechanism, including Naïve Bayes (NB), k-nearest neighbor (kNN), and support vector machine (SVM).

#### 3.3.1. Feature Analysis and Selection

We investigate two feature sets for significant change spotting. The first feature set contains traditionally used statistical features. Since the total number of samples within each significant series candidate is much smaller, complex statistical features, such as skewness, kurtosis, and spectral entropy may not be reliably calculated. Therefore, we only consider statistical features that can be reliably calculated at a primitive level. The statistical features are listed in Table 2.

The second set of features are called physical features, which are derived based on the physical parameters of an upper limb functionality assessment [46,47]. Most of these physical features can provide accurate and objective information about the movement quality and smoothness for human m. Table 3 lists the physical features including in this work.

Feature selection for a machine learning-based classifier is the critical process during significant change spotting. Selecting the suitable features can reduce the computational effort of the classification process, and improve the classifier performance. Furthermore, the selected features can provide causal relationships between features and classes for researchers. The sequential forward selection approach is utilized to select relevant features during feature selection. The method is employed by repeatedly adding features to the feature vector with the currently best quality.
**Algorithm 1**: Candidate search algorithm for significant seriesInput:A sequence of data D $=\left\{d_{i}|i=1,2,\ldots ,N_{D}\right\}$, a set of events $E=\left\{e_{i}|i=N_{E}\right\}$,the numbers of events $N_{E}=0$Output:A series of candidates for significant series $S=\left\{s_{i}|i=N_{S}\right\}$, $N_{S}$ is the totalnumber of candidates for significant series1:$\theta_{thres}$ ← calculateMean(X) /* Threshold definition for event detection2:for i from 1 to $N_{D}$ do  /* Detect the event for the set of candidates3: if $d_{i}\;-\theta_{thres}\langle 0\;\mathrm{and}\;d_{i+1}\;-\theta_{thres}\rangle 0\;then$4:  $N_{E}=N_{E}+1$
5:  $e_{N_{E}}\leftarrow \left(d_{i},d_{i+1}\right)$
  $E\leftarrow e_{N_{E}}$
6: end if7:end for8:for $i=1\;\mathrm{to}\;N_{E}\;do$   /* Candidate search for the significant series9: $\left(d_{j},d_{k}\right)\leftarrow \mathrm{getCurrentSet}\left(e_{i}\right)$
10: $d_{c_{max}}\leftarrow \mathrm{extendLocalMaximum}\left(d_{j}\right)$
11: $d_{c_{min}}\leftarrow \mathrm{extendLocalMaximum}\left(d_{k}\right)$
12: $S\leftarrow \mathrm{getCandidate}\left(d_{c_{min}},d_{c_{max}}\right)$
13:end for14:return S

#### 3.3.2. Spotting and Classification

There are various classification approaches that have been developed from simple threshold-based, to more advanced, machine learning algorithms. Considering the computational complexity, classification performance, target activity, and type of application, the selection of appropriate machine learning-based approaches is a critical component [48,49]. While this work focuses on the segmentation process belonging to the early process of activity monitoring system, some powerful machine learning-classifiers with high computational complexity are not included, such as neural networks. Therefore, the following three typical types of machine learning-based classifiers are utilized and introduced.

Naive Bayes (NB) classifier: NB is a statistical approach to estimate the possibility of a given instance. The Naïve Bayes probabilistic model makes assumptions that instances have possibilities relating to an independent class. The NB classifier selects a class label that has the greatest probability based on the observed data, using Bayes rule and assuming conditional independence. This work uses a Bayesian classifier where Gaussian distributions were used to model the priors of the classes.

K-nearest neighbor (kNN): The kNN algorithm is one of the most popular algorithms for machine learning. It is a supervised learning algorithm where the result of new instance query is classified based on the majority of a kNN category. The kNN algorithm is amongst the most basic of all machine learning algorithms: a target is assigned by a majority vote of its k-nearest neighbors (k is a positive integer, typically small). The kNN classifier uses the kNN algorithm as the prediction value of the new query instance. The classifier does not use any model to fit and is only based on memory. Many researchers have proved that the kNN classifier accomplishes results with good performance in different applications. The kNN classifier utilized in this work uses a Euclidean formula to calculate the distance between two points in multidimensional space, and classifies test data with k=5. 

Support vector machine (SVM): SVM is one of the standard tools for machine learning and data mining. SVM aims to define the hyperplane that maximizes the distance between the two parallel hyperplanes. Intuitively, the larger margin means a lower generalization error of the classifier. SVM identifies a function that maximizes the distance to data points in both classes. SVM classifiers are applied with a RBF (radial basis function) kernel function in this work.

## 4. Results and Data Analysis

### 4.1. Evaluation Methodology

The leave-one-out cross-validation (LOOCV) is adopted in this work. LOOCV uses one segment as the testing set and the remaining set as the training set. There are a total of k segments created according to the total days performed by the subject. For example, there are five segments while he subject performed five cleaning tasks on five different days. One segment is used as the testing dataset while the rest of the (k −1) segments are used as the training dataset, and repeated k times to evaluate the proposed segmentation approach.

As shown in the Figure 6, several tags are designed to validate the results of the proposed approach. While spotting is the main target, there are several metrics to measure performance of the proposed significant change spotting for motion segmentation. The recognition performance is measured in precision, recall, and F1-score commonly used for information retrieval assessments. These evaluation metrics are defined in Equations (6)–(8). Precision indicates the percentage of times that a recognition result made by the system is correct. Recall means the percentage of times that an activity performed by a user is detected by the system. F_1_-score is an integrated measure that combines both.

(6) $$\mathrm{Recall}=\frac{\mathrm{TP}}{\mathrm{TP}+\mathrm{FN}}$$

(7) $$\mathrm{Precision}=\frac{\mathrm{TP}}{\mathrm{TP}+\mathrm{FP}}$$

(8) $$F_{1}\text{-}\mathrm{score}=\frac{2\;\cdot \;\mathrm{Recall}\;\cdot \;\mathrm{Precision}}{\mathrm{Recall}+\mathrm{Precision}}$$

### 4.2. Search and Spotting Results

**Figure 7:**
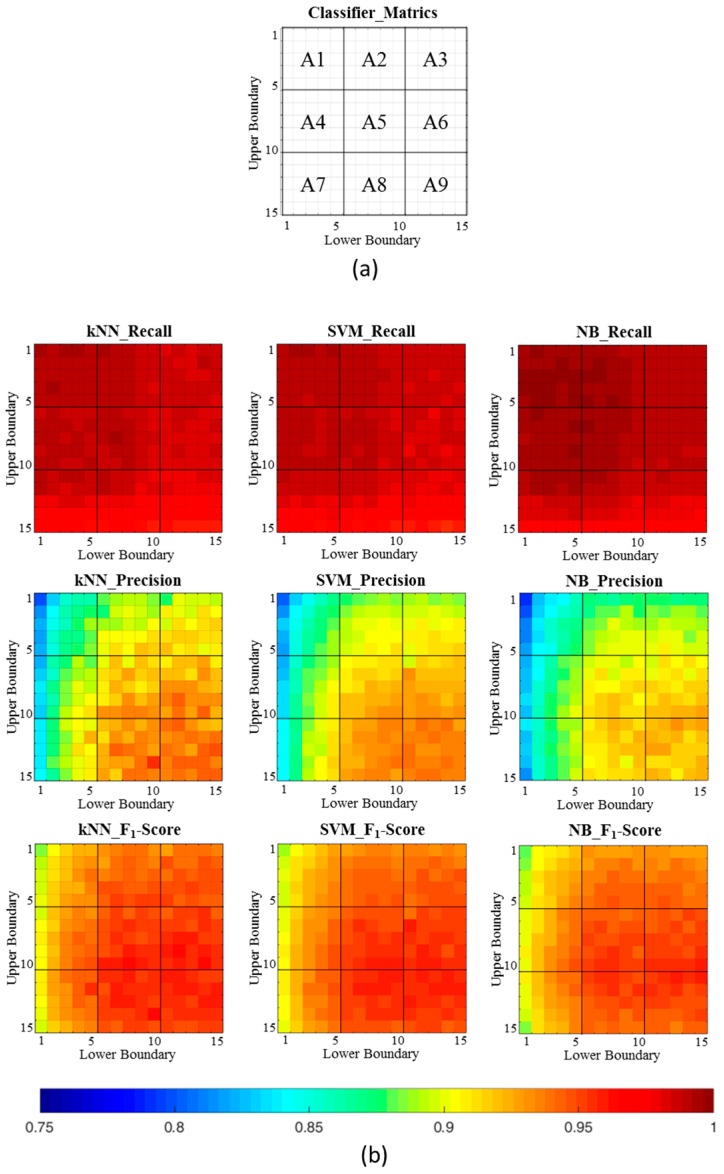
Overall performance comparison of different classifiers. (**a**) An example of performance divided into nine areas. Each area can be defined as the average performance of 25 pairwise combinations of different upper and lower boundaries. (**b**) Pairwise combinations of different upper and lower boundaries (samples) are shown in each performance metric.

Figure 7 shows the overall performance metrics of the kNN, SVM, and NB classifiers, however, which is not easy to express and expound the proposed significant change spotting approach of human motion segmentation for cleaning task monitoring. Therefore, each performance metric is split into nine areas in row major order, and each area can be defined as the average performance of 25 pairwise combinations of different upper and lower boundaries ($\alpha_{\mathrm{upper}}$, $\alpha_{\mathrm{lower}}$). For example, area A4 contains the pairwise combinations of the upper boundary from 5 to 10, the lower boundary from 1 to 5, and a total of 25 pairwise combinations.

Table 4 shows nine areas of performance comparisons of different classifiers. The average performance of the three utilized classifiers with the highest recall (99.27 ± 0.25) is located in the A1 area, the highest precision (93.1 ± 0.76) is located in A9, and the highest F1-score (95.26 ± 0.25) is located in A9.

**Figure 8:**
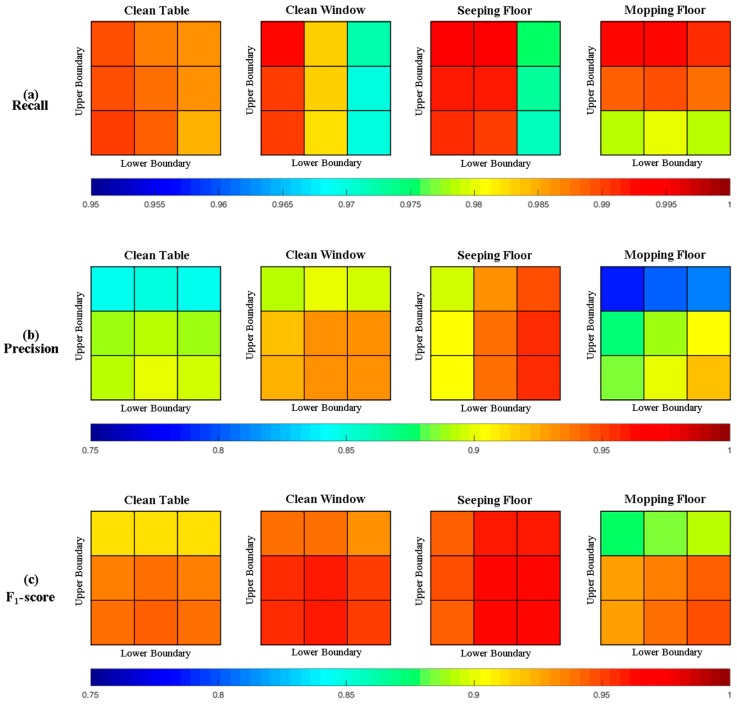
Average performance of three classifiers for four cleaning tasks in (**a**) recall; (**b**) precision; and (**c**) F1-score.

Since wearable sensors are sensitive to motion performance, this work explores whether it is feasible to build a general segmentation model for four different cleaning tasks in a laboratory. Figure 8 shows the proposed significant change spotting approach for four cleaning tasks in recall, precision, and F_1_-score, respectively. Obviously, the proposed approach for each cleaning tasks has its suitable areas of upper and lower boundary. Firstly, for the performance of floor sweeping, the areas with the same upper boundary show a downward trend in recall as the lower boundary increases, but the performance in precision is the inverse. A similar situation also happened in the floor mopping task, where the areas with the same lower boundary show a downward trend in recall as the upper boundary increases, but the performance in precision is the inverse. Secondly, for the performance of cleaning the table, the difference between areas with the highest recall (99.03% in A9) and with the lowest recall (98.49% in A7) is only 0.54%, but the difference between areas with the highest precision (90.19% in A8) and with the lowest precision (84.69% in A3) is 5.51%. Thirdly, the difference between areas with the highest recall (99.03% in A9) and with the lowest recall (98.49% in A7) in four cleaning tasks are not above 2.26%. Therefore, the performance of the proposed segmentation in F_1_-score really depends on the performance in precision. 

Table 5 shows the pairwise combination with the best performance in recall, precision, and F_1_-score for four cleaning tasks. Firstly, for the pairwise combination with the best performance in recall, the pairwise combination with low $\alpha_{\mathrm{lower}}$ has the best performance. It also shows that most of the pairwise comparisons for the four cleaning tasks have the best performance with high $\alpha_{\mathrm{upper}}$ and $\alpha_{\mathrm{lower}}$ precision and recall. As shown, the NB classifier has the best performance in recall for the four cleaning tasks. However, the performance of each cleaning task in precision and F_1_-score has its appropriate classifier. Overall, the best performance in the F_1_-score of the pairwise combinations is (9, 19), which achieves 96.41% in the F_1_-score by using the SVM classifier. 

Table 6 shows the selected features of each dominant axis in descending order. The top four selected features in all axes are the same, but with different order. *RMS* and Movement Time are only selected once, and Maximum is selected when the dominant axis is the X or Y axis. The features STD and Mean, Minimum, and Maximum are selected amongst the three axes.

## 5. Discussion

Overall, the proposed segmentation approach using kNN and SVM have better performance than using NB. Three metric performance differences between the two classifiers are only 0.03% to 0.12%. However, kNN requires storage and access to all of the training data. Thus, kNN is less scalable for a large dataset and less practical for wearable sensors given their limited storage. Therefore, SVM is more suitable for implementation on the wearable sensors.

Based on our results, the most challenging cleaning tasks for the proposed segmentation approach is “mop floor”. Since the mopping motions are composed of fragmental parts, the significant changes during mop floor is invisible to the classifiers. This outcome varies in intensity amongst individuals, which leads the classifiers to have poor performance in recall across four cleaning tasks. However, the F_1_-score is still adequate across all participants, and even using the NB classifier achieves an average of 91.27% in the A1 area, at least.

As shown in Figure 8, the proposed segmentation with the higher $\alpha_{\mathrm{upper}}$ and $\alpha_{\mathrm{lower}}$ shows the non-obvious downward trend in recall. The significant improvement in precision is shown as $\alpha_{\mathrm{upper}}$ and $\alpha_{\mathrm{lower}}$ increase, which leads the performance in precision as the main effect on the F1-score. One reason for these phenomena is that the effect of muscle vibration causing significant changes are not monotonous. Therefore, the low $\alpha_{\mathrm{upper}}$ and $\alpha_{\mathrm{lower}}$ of the extension function is unable to find the true significant changes in the significant series search. Only parts of the significant change are found, which easily confuses the classifier and finally leads to misclassification. Therefore, the ability of the proposed approach to include true significant changes is considered as the critical factor for the results, even with the powerful classifier.

As shown in the Table 6, there is a strong commonality in the top four selected features, including Maximum, Minimum, Mean, and STD, when the dominant axes are the X and Y axes. Such strong commonality reflects and fulfills the attribute of the proposed significant change approach. The different ranked order also reveals the different consideration of each axis. Specifically, the features peak number and jerk are not selected in this work, which have similar results with previous studies [46]. This is because target activities are performed smoothly by the healthy subject. Further study should be carried out to deal with different situations, especially for a subject with a functional disability of an upper limb.

We observed a large amount of individual variability in cleaning styles. Various forms of the gesture of holding the utensil during cleaning tasks were observed, even though the subjects were asked to perform on the same target. Additionally, some subjects perform cleaning tasks continuously without any temporary stop, while some others take a temporary stop during the cleaning tasks to check the state of the cleaned target. These could be attributed to an individual’s own cleaning style.

In this study we focus on building an effective segmentation approach for each observed cleaning style; all cleaning gestures are given different labels. However, more cleaning tasks and other ADLs are not considered in this work, such as sink cleaning in the kitchen and bathroom, and toilet cleaning. Without any question, this posed an additional challenge to the classification task. In future work, ambient sensors are planned to be employed in order to reflect greater interaction information between body motion and context, such as room transition and the use of utensils, which might contribute significantly to the motion segmentation of cleaning tasks.

Despite the importance of high recall, precision, and F_1_-score, the practical problem that still needs to be addressed depends largely on the application domain. In contrast to physical activity monitoring used for energy consumption estimation, gesture performance measurement during ADLs are particular challenging. This is because the requirements of activity information are different. In order to detect early changes in aging, which reflects a decline in the ability of independently perform ADLs, the requirement of continuously and fine-grained activities information collection is essential. The experimental results show that the proposed approach can tackle many technical challenges, such as continuous signal segmentation, individual variability, and activity ambiguity. The proposed significant spotting-based segmentation approach can play an important role in activity monitoring, and provide an efficient approach for continuous cleaning task monitoring.

## 6. Conclusions

In order to develop an automatic ADL monitor for the assessment of daily functionality in remote health monitoring applications, especially in early detection of a change in aging, the automatic ADL monitoring requires reliable ADL information on a fine-grained level. In this work, we proposed a significant change spotting mechanism for periodic human motion segmentation during performance of cleaning tasks. A novel approach is proposed based on the search for significant change of gestures, which can deal with critical technical issues, such as continuous data segmentation, individual variance, and category ambiguity. Three different classification algorithms are utilized to validate the significant change candidate through machine learning-based spotting, including a Support Vector Machine (SVM), k-Nearest Neighbors (kNN), and Naïve Bayesian (NB) algorithm. The experimental results have demonstrated that the proposed segmentation approach achieves a high F1-score. The proposed approach can be a part of automatic ADL monitoring activity recognition for the fine-grained information of cleaning tasks, such as the number of cleaning gestures performed, and the duration of each cleaning task. Such fine-grained information of cleaning tasks has the potential to assist caregivers and clinicians to identify a decline in the ability of independently performing ADLs in a quantitative and objective way. In future work, cleaning task monitoring based on the proposed segmentation approach will be presented and designed in the real world. The study will collect the data from the elderly to assess the ability of the proposed approach. Furthermore, a discussion about, and a comparison of, filters, machine learning algorithms, and segmentation approaches are planned to be investigated.

## Figures and Tables

**Table 1:** The description of the cleaning tasks.

Activity No.	Cleaning Tasks	Description
1	Clean Window	Window wiping with a rag
2	Clean Table	Table wiping with a rag
3	Sweep Floor	Floor sweeping with a broom
4	Mop Floor	Floor sweeping with a mop

**Table 2:** Statistical features calculation and description.

No.	Statistical Feature	Description
1	Maximum	The average value of the signal over the significant series
2	Minimum	The maximum value of the signal over the significant series
3	Mean	The minimum value of the signal over the significant series
4	Standard Deviation (STD)	Measure of the spread of the signal over the significant series
5	Root Mean Square (RMS)	The quadratic mean value of the signal over the significant series
6	Range	The difference between the maximum and minimum values over the significant series

**Table 3:** Physical features calculation and description.

No.	Physical Feature	Description
7	Movement time	Measure of the time required over the significant series
8	Peak number	Gradient analysis of the signal over the significant series
9	Jerk metric	Quality measure of smoothness over the significant series

**Table 4:** The overall performance comparison of different classifiers for four cleaning tasks.

Measure Metric (%)	Classifier	Nine Areas								
		A1	A2	A3	A4	A5	A6	A7	A8	A9
Recall	kNN	99.09	98.88	98.52	98.84	98.75	98.36	97.93	97.71	97.29
	SVM	99.09	98.85	98.55	98.92	98.77	98.37	97.92	97.69	97.29
	NB	99.62	99.40	99.03	99.46	99.30	98.95	98.53	98.32	97.99
	mean (±Std)	99.27 (±0.25)	99.04 (±0.25)	98.7 (±0.24)	99.08 (±0.28)	98.94 (±0.26)	98.56 (±0.28)	98.13 (±0.29)	97.91 (±0.29)	97.53 (±0.33)
Precision	kNN	85.45	89.83	90.55	87.41	91.96	92.66	88.28	93.08	93.70
	SVM	85.41	89.60	90.14	87.25	91.83	92.33	88.25	93.01	93.58
	NB	84.23	88.70	88.93	86.37	90.78	91.35	86.65	91.37	92.03
	mean (±Std)	85.03 (±0.56)	89.37 (±0.49)	89.87 (±0.69)	87.01 (±0.46)	91.52 (±0.53)	92.11 (±0.56)	87.73 (±0.76)	92.49 (±0.79)	93.1 (±0.76)
F1-score	kNN	91.75	94.13	94.37	92.76	95.23	95.42	92.83	95.33	95.46
	SVM	91.73	94.00	94.16	92.70	95.17	95.25	92.81	95.29	95.40
	NB	91.27	93.74	93.71	92.44	94.85	95.00	92.19	94.71	94.91
	Mean (±Std)	91.58 (±0.22)	93.96 (±0.16)	94.08 (±0.28)	92.63 (±0.14)	95.08 (±0.17)	95.22 (±0.18)	92.61 (±0.3)	95.11 (±0.28)	95.26 (±0.25)

**Table 5:** The best performance of the proposed segmentation approach for four cleaning tasks.

Cleaning Task	Measure Metric	(%)	($\alpha_{\mathrm{upper}}$, $\alpha_{\mathrm{lower}}$)	Selected Classifier
Clean Table	Recall	99.79	[1, 1]	NB
	Precision	94.86	[11, 14]	kNN
	F1-score	96.82	[11, 14]	kNN
Clean Window	Recall	99.86	[1, 2]	NB
	Precision	97.73	[14, 10]	SVM
	F1-score	97.39	[10, 8]	SVM
Sweep Floor	Recall	99.92	[5, 2]	NB
	Precision	98.07	[13, 7]	SVM
	F1-score	98.28	[10, 14]	SVM
Mop Floor	Recall	99.81	[12, 5]	NB
	Precision	95.82	[14, 10]	NB
	F1-score	96.76	[14, 10]	NB
Overall	Recall	99.74	[5, 2]	NB
	Precision	95.33	[14, 10]	SVM
	F1-score	96.41	[9, 12]	SVM

**Table 6:** Selected features of each dominant axis in ranked order for the significant change spotting.

Dominant Axis	Ranked Feature
X-axis	Maximum, Minimum, Mean, STD, Range, RMS, Movement Time
Y-axis	Maximum, Mean, Minimum, STD, Range
Z-axis	Minimum, STD, Mean, Maximum
